# A Nanoparticle-Based Label-Free Sensor for Screening the Relative Antioxidant Capacity of Hydrosoluble Plant Extracts

**DOI:** 10.3390/s19030590

**Published:** 2019-01-30

**Authors:** Melinda David, Adrian Şerban, Claudia V. Popa, Monica Florescu

**Affiliations:** 1 Faculty of Medicine, Transilvania University of Braşov, Colina Universităţii nr 1, Corp C, room CI30, 500068 Braşov, Romania; melinda.david@unitbv.ro (M.D.); adrianserban1994@yahoo.ro (A.Ş.); 2 Department of Organic Chemistry, Biochemistry and Catalysis, University of Bucharest, Sos. Panduri 90-92, 050657 Bucharest, Romania; popa_vali2006@yahoo.com

**Keywords:** label-free electrochemical sensors, relative antioxidant capacity, gold nanoparticles, ROS scavenging

## Abstract

One of the most important aspects of the detection of antioxidant compounds is developing a fast screening method. The screening of the overall relative antioxidant capacity (RAC) of several Romanian hydrosoluble plant extracts is the focus of this work. This is important because of the presence of increasing levels of reactive oxygen species (such as H_2_O_2_) generates oxidative stress in the human body. The consequences are a large number of medical conditions that can be helped by a larger consumption of plant extracts as food supplements, which do not necessarily contain the specified antioxidant contents. By exploiting the catalytic properties of gold nanoparticles, a specific and sensitive nanoparticle-based label-free electrochemical sensor was developed, where the working parameters were optimized for RAC screening of hydrosoluble plant extracts. First, electrochemical measurements (cyclic voltammetry and amperometry) were used to characterize different nanoparticle-based sensors, revealing the best performance of gold nanoparticle-based sensors, obtaining a RAC of 98% for lavender extracts. The sensing principle is based on the quenching effect of antioxidants for H_2_O_2_ amperometric detection, where the decrease in electrical signal suggests an increasing antioxidant capacity. The obtained results were expressed in terms of ascorbic acid and Trolox equivalents in order to be able to correlate our results with classical methods like chemiluminescence and UV-Vis spectrophotometry, where a correlation coefficient of 0.907 was achieved, suggesting a good correlation between electrochemistry and spectrophotometry. Considering these results, the optimized gold nanoparticle-based label-free sensor can be used as a simple, rapid alternative towards classical methods for relative antioxidant capacity detection of hydrosoluble plant extracts.

## 1. Introduction

Free radicals are molecules containing oxygen or nitrogen, and one or more unpaired electrons, making them very reactive. In organisms, they are referred to as reactive oxygen/nitrogen species (ROS/RNS) and are generated in metabolic and physiological processes. Some of the most important ROS are the radicals of hydroxyl, superoxide anion, peroxynitrite or hydrogen peroxide (H_2_O_2_) and singlet oxygen [1]. The imbalance between ROS and antioxidant molecules (AOx) leads to oxidative stress (OS), which has been correlated to various disorders and medical conditions caused by damage to healthy cells, DNA and protein molecules. Oxidative stress will then initiate lipid peroxidation [2]. DNA mutations and alterations induced by OS were proven to be involved in cancer pathogenesis and proliferation. Furthermore, an abnormal oxidation status in the human body has been linked with chronic diseases such as diabetes, cardiovascular and neurological diseases [3].

AOx present in organisms have the major role to scavenge and neutralize ROS and their precursors, acting as defense systems [4]. Based on their activity, biological AOx can be categorized in two classes: enzymatic and non-enzymatic. Enzymatic AOx are superoxide dismutase, catalase, glutathione peroxidase, etc. while the non-enzymatic antioxidants could be antioxidant enzyme cofactors (selenium-Se, coenzyme Q10 etc.) [5]. AOx can deactivate radicals by two major mechanisms: hydrogen atom transfer (HAT) and single electron transfer (SET) [6]. The end results are the same, regardless of the mechanism; however, kinetics and potential for side reactions differ. Both mechanisms almost always occur together in all samples, with the balance determined by pH and antioxidant structure. HAT-based methods measure the classical ability of an antioxidant to quench free radicals by hydrogen donation (AH = any H donor), as shown in Equation (1):

X^•^ + AH → XH + A^•^ (1)

SET- based methods detect the ability of a potential antioxidant to transfer one electron resulting in AH^•+^, in order to reduce any compound, followed by deprotonation in solution to give the corresponding very stable A^•^ radical, as shown in the following two equations:

X^•^ + AH → X^−^ + AH^•+^ (2)

AH^•+^ + H_2_O ↔ A^•^ + H_3_O^+^ (3)

The presence of AOx can be determined by measuring their antioxidant capacity (AC). The capacity of the human antioxidant system can hereby be divided into two main groups: AC of the cell, which is attributed to the enzymatic AOx, and the AC of plasma, which is mostly associated with AOx of dietary origin [7]. Many fruits, vegetables and medicinal herbs (e.g., sea buckthorn and lavender) play an important role in human health due to their high content in AOx molecules. The main classes of dietary AOx are vitamins, carotenoids and polyphenols. Vitamin C is known to reduce the incidence of chronic and degenerative diseases and can be found in sea buckthorn berries together with carotenoids [8,9]. The activity of polyphenols prevents cardiovascular diseases, and shows important anti-inflammatory and anti-viral properties [10]. Several classes of polyphenolic compounds can be found in lavender [11,12], while a total of 322.2 mg/kg flavonol content was found in grapes [13,14] and a much higher content of phenolics ranging between 65 to 73 g/kg, where flavonols are the major compound, was found in walnuts [15]. In this work, for comparison, spectrophotometry measurements were performed on walnuts and grapes.

Plant extracts are among the most studied dietary supplements due to their high antioxidant capacity. Up to this point, a fast screening method of the AC is not yet developed. The complex chemical composition of plant extracts can be determined using classical, elaborate and time-consuming methods such as: luminescence-chemiluminescence [16], chromatographic methods such as high performance liquid chromatography [17], or spectroscopic methods like Fourier transform infrared with complementary UV-Vis spectra [18]. Electrochemical methods were also investigated and have proven to be more rapid. A chronoamperometric study was performed by Brainina et al. [19], where the total antioxidant activity was correlated to the oxidation current of a mediator (potassium ferrocyanide K_4_[Fe(CN)_6_]) which is formed in the reduction of potassium ferricyanide (K_3_[Fe(CN)_6_]) in the presence of AOx. Another amperometric xanthine oxidase (XOD)- based sensor was developed by Bucur et al. [20]. The quantification of the analytical signal was based on the detection of H_2_O_2_, where xanthine and ascorbic acid were considered antioxidants. A further cell-based biosensor was proposed by Ge et al. where H_2_O_2_ was generated by cell stimulation and subsequently monitored using differential pulse voltammetry. The relative antioxidant capacity of *L. plantarum* extracts, incubated in cells, was expressed in terms of their inhibitory effect on the generation of H_2_O_2_ [21].

All the above biosensors have an elaborated architecture making use of several chemicals and contain enzymes or cells, which have a limited lifetime; therefore, we propose the use of a simple system, based on sensors modified with nanoparticles, towards AC detection, as illustrated in the graphical abstract. In our previous work [22] functionalized graphene and carbon nanotubes (CNTs) were successfully used by increasing the specific capacitance of the carbon nanomaterials and enabling rapid electrolyte ion transport. In this work, several nanoparticle-based screen printed electrodes (SPEs) were compared and their working parameters optimized. Electrochemical measurements such as cyclic voltammetry (CV) and amperometric detection were used to evaluate sensor performance and the relative antioxidant capacity of the plant extracts. According to Annapandian et al. [23] the total antioxidant capacity of extracts can be expressed as ascorbic acid (AA) equivalents (mg/100 mL extract). Achieving our main aim, we have studied the scavenging capacity of AOx present in the extracts towards H_2_O_2_, where the RAC was expressed using a standard calibration plot of AA. In order to be able to correlate our results with the classical method of chemiluminescence and UV-vis spectrophotometry, the RAC was also expressed in terms of Trolox equivalents. Scanning electron microscopy (SEM) was used to gain insights into the physical appearance and the morphology of the plant extracts obtained with different extraction methods.

## 2. Materials and Methods

### 2.1. Reagents

All reagents used were of analytical grade. H_2_O_2_ (30%, MW = 34.02 g/mol) and ethylenediaminetetraacetic acid disodium salt (EDTA) were purchased from SC. NORDIC INVEST SRL. (Cluj-Napoca, Romania) Ascorbic acid and Trolox (6-hydroxy-2,5,7,8-tetramethylchroman-2-carboxylic acid; 97%, MW = 250.29 g/mol) were purchsed from Sigma-Aldrich (Steinheim, Germany). Glycerin, propylene glycol and ethanol were from S.C. Chemical Company S.A. (Iaşi, Romania). Boric acid and cobalt(II) chloride hexahydrate were from Reactivul (Bucureşti, Romania); 5-amino-2,3-dihydro- naphtalazine-1,4-dione (luminol) was from Fluka BioChemie (Bratislava, Slovakia) and gallic acid monohydrate was purchased from Riedel-deHaën (Bratislava, Slovakia).

Electrochemical and spectrophotometric measurements were performed in pH 7.0 sodium phosphate buffer (NaPB, 0.1 M). Millipore Milli-Q nanopure water (resistivity ≥ 18 MΩ cm) was used for the preparation of all solutions. All experiments were performed at room temperature (22 ± 1 °C) and no special conditions for keeping the SPE in between measurements were needed.

The extracts were obtained from Romanian sea buckthorn berries, lavender flowers, dried walnuts and grape skin and seeds. Water of two different pH used for the hydrosoluble extracts was obtained using a Leveluk Kangen water apparatus and they were denoted as: water “a” (pH  =  2.5) and water “b” (pH  =  9.5). Ethanol, glycerol and propylene glycol were of pharmaceutical grade.

### 2.2. Preparation of Plant Extracts

Two extraction techniques, ultrasound-assisted extraction and pressure enhanced solvent extraction at 6.7 bar were used for a comparative study of the active compounds from different plants. The lavender flowers (Lf), sea buckthorn berries (Hf), dry walnuts (Js) and selected grape skin and seeds (Vp) were dried in a dryer (Miraco, MIRACO SRL. Bucureşti, Romania) with controlled temperature (50–60 °C) and humidity. The dried flowers, seeds, nuts and berries were crushed in a type TP2 mill (MIRACO SRL. Bucureşti, Romania) and sieved using a stainless sieve with 4 mm size.

Ultrasound-assisted extraction (US) was performed using a Sonomatic Langford bath (EJ Electronics Limited, Bromsgrove, UK) with a controlled temperature at 35 °C. Rapid extraction was performed in a Timatic (T) Micro C (Tecnolab, S.R.L., Spello, Italy) at 35 °C at 6.7 bar. For all extractions, a mixture of dried plants and corresponding extracting solvent in proportion of 1 g/10 mL have been used. As extracting solvent aqueous solutions were prepared by separately mixing water “a” and water “b” with the corresponding organic solvent of ethanol (A), glycerol (G) or propylene glycol (Pg) in a volume ration of 1:1, as tabulated in Table 1. Dry walnuts and grape extracts were only used for spectrophotometric measurements and their data will be presented later on.

### 2.3. Instrumentation

All electrochemical measurements were carried out in a conventional electrochemical cell. Screen-printed carbon electrodes were printed on a ceramic substrate with three silver electrical contacts for the nanoparticle modified working electrode, a carbon auxiliary electrode and silver reference electrode (DropSens, Llanera (Asturias), Spain). The working electrodes having a geometrical surface area of 0.1256 cm^2^ were modified with: single walled carbon nanotubes and denoted CNT, graphene denoted G, or gold nanoparticles denoted AuNP. The SPEs were used as received. Voltammetric and amperometric measurements were performed by using a PalmSens3 electrochemical sensor interface (Palm Instruments BV, Houten, The Netherlands) controlled with PSTrace 5.2 software.

Scanning electron micrographs of the plant extracts were taken using a Hitachi SU-70 microscope (Hitachi High Tehnologies, Tokyo, Japan)). The conditions for morphological investigations of the extracts were: field-free mode, 5 kV accelerating voltage, and working distance in the range of 13.7–19.4 mm.

All chemiluminescence (CL) measurements were carried out with a 20/20^n^ luminometer (Turner Biosystems Inc., Sunnyvalle, CA, USA) coupled to a computer, so that the software (SIS for 2020h) could be used to record the light intensity as a relative luminescence unit (RLU) as a function of time (in second).

Absorbance measurements were recorded using a FLAME-S spectrometer (Ocean Optics Inc., Largo, FL, USA) preconfigured for 200–1050 nm. The balanced DH-2000-BAL deuterium tungsten halogen light source provides illumination from 230–2500 nm. Data can be visualized and analysed by the corresponding Ocean View 1.6.3 software. The path length for absorption spectra was 1 cm. Statistical analyses were performed using paired *t*-tests and the correlation coefficient was calculated using Pearson’s correlation.

## 3. Results and Discussions

### 3.1. Electrochemical Sensor Characterization and Optimization

Electrochemical measurements by means of cyclic voltammetry and amperometry were carried out in order to evaluate the best electron-conducting material. Graphene is known to have a huge surface area, showing excellent electrical conductivity, which remains stable over a vast range of temperatures [24]. CNTs are the most used in electrochemical applications as they show great electrical conductivity [25], while AuNPs have the ability of electron transfer among different electroactive (biological) species, due to their high electroactive surface area and fine dimensions [26]. Among the mentioned nanoparticles, AuNPs have been proven to be the most adequate for antioxidant capacity detection, as shown below.

**Figure 1:**
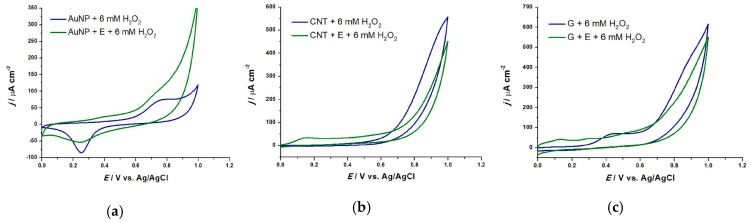
CV in 0.1 M NaPB, pH 7.0, in the presence and absence of the extract (E), with 6 mM H_2_O_2_ addition, v = 50 mV s^−1^ for: (**a**) AuNP, (**b**) CNT and (**c**) graphene (G).

Using CV, three parameters of the reducing behaviour of AOx can be monitored: the anodic peak current (I_a_) with the oxidation potential (E_pa_) and the anodic area (Q) [27]. In this work H_2_O_2_ is used as ROS, whose inhibiting (quenching) effect upon AOx present in the analyzed plant extracts is monitored. First, the response of 6 mM H_2_O_2_ in 0.1 M NaPB pH = 7.0 of all nanoparticle-based sensors was monitored by CV, scan rate 50 mV s^−1^. In the following step, the quenching capacity of an extract diluted in 0.1 M NaPB pH = 7.0 at a particular extract-to-buffer electrolyte ratio, in the presence of the same H_2_O_2_ concentration was determined, where a decrease of the sensor response was expected. We noticed the highest decrease in the case of AuNP modified SPE as shown in Figure 1a where at 0.22 V the reduction peak decreases by 39% in the presence of an extract. We attributed this behavior to the catalytic properties of AuNP, while, in the case of CNTs and graphene, the decrease is of 19% and 20% (Figure 1b,c). In the presence of an extract, cyclic voltammograms reveal anodic peaks at low redox potentials (below 0.45 V), that are known to occur for the compounds with significant antioxidant activity [28]. A comparison between the nanoparticle modified SPE and a bare SPE was also made by recording cyclic voltammograms in the same conditions. This offers an insight into the changes of the capacitance values for each nanoparticle type. Capacitance values were calculated in the non-faradaic region at 0.55 V (Supplementary Materials). The graphene-modified sensor shows a high capacitance value of 1.23 mF cm^−2^, highlighting the larger surface area of graphene with excellent conducting properties. However, it does not show any catalytic properties in the presence of AOx, to enhance their quenching properties towards H_2_O_2_. The AuNP and CNT-modified sensors show similar values (0.34 and 0.30 mF cm^−2^ respectively), thus in the presence of an extract, only AuNP-modified sensor shows an improved catalytic behavior of the AOx compounds. As expected, the smallest value (0.03 mF cm^−2^) was calculated for the bare SPE.

**Figure 2:**
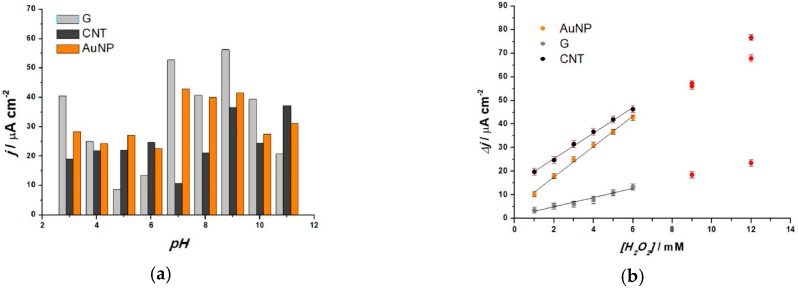
(**a**) pH study-data from CV in 0.1 M NaPB with extract and 6 mM H_2_O_2_ addition, v = 50 mV s^−1^ and (**b**) Calibration plots recorded at 0.55 V vs. Ag/AgCl in 0.1 M NaPB with extract, pH = 7.0.

A pH study was also conducted using CV, in order to determine the best performing nanoparticle type for H_2_O_2_ detection in the presence of extracts and the results obtained are shown in Figure 2a. The pH values ranged from 3 to 11. The histogram represents the current density value in the anodic region corresponding to the H_2_O_2_ oxidation potential of 0.55 V for all nanoparticle-based sensors as a function of pH. A fixed potential was chosen to avoid the oxidation peaks generated by extracts alone in the buffer. Even though higher current densities were recorded in the case of graphene, the sensor with AuNPs has a more stable behavior, while that with CNTs shows the poorest performance. The optimum pH value, 7.0, was chosen to be the one for which the AuNP-sensor had the highest response (43.05 µA cm^−2^), and it was used in all further experiments. For further nanoparticle evaluation, fixed potential amperometry measurements (n = 3) were performed for all three sensors in 0.1 M NaPB, at 0.55 V vs. Ag/AgCl, upon addition of increased H_2_O_2_ concentrations, with the corresponding calibration plots shown in Figure 2b. The lowest sensitivity was obtained for the graphene-modified sensor with a value of 1.94 ± 0.18 µA cm^−2^ mM^−1^, while the best sensitivity was obtained at the AuNP-modified sensor at 6.43 ± 0.2 µA cm^−2^ mM^−1^ (R^2^ = 0.995). From all above data, we chose the AuNP modified SPE, which showed the best inhibiting behavior of the extract upon H_2_O_2_, which was therefore used in all following measurements.

The optimal dilution of the extract was also tested using amperometric measurements recorded at 0.55 V vs. Ag/AgCl at AuNP-modified SPE in 0.1 M NaPB (pH 7.0) and three extract dilutions using the extract:buffer ratio as follows: 1 : 20, 1 : 40 and 1 : 60. The corresponding calibration plots were represented for increasing H_2_O_2_ concentrations (Supplementary Materials), while a low detection limit and a high sensitivity are of interest. Herewith, the best results were obtained for the ratio 1:40, with a sensitivity of 11.37 µA cm^−2^ mM^−1^, R^2^ = 0.995 and a detection limit of 0.75 mM for a linear range between 2–10 mM H_2_O_2_. Even though the sensitivity for the ratio 1:60 was higher, the detection limit was higher, while the linear range was lower.

**Figure 3:**
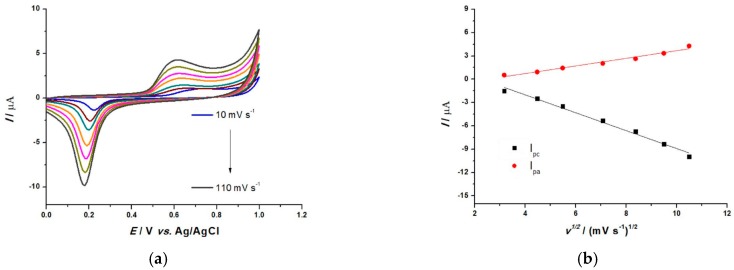
(**a**) Cyclic voltammograms in 0.1 M NaPB, pH = 7.0 at AuNP-modified sensor for different scan rates: 10, 20, 30, 50, 70, 90 and 110 mV s^−1^; (**b**) Plots of both peak currents I_pa_ and I_pc_ vs. the square root of the scan rate.

The AuNP based sensor was further characterized by means of the electrochemical mechanism of the AuNP, highlighting the relationship between the scan rate and the peak current during CV measurements in 0.1 M NaPB (pH 7.0), and the results obtained are displayed in Figure 3. AuNPs have been proven to act as a catalyst [29]. There is a considerable increase of the current intensity in the buffer with increasing scan rate (from 0.59 to 4.31 µA in the anodic region and from −1.33 to −9.9 µA in the cathodic region), suggesting an increase of the electron transfer rate. The oxidation peak potential at 0.6 V is almost unchanged, exhibiting a linear relationship with the square root of the scan rates over the range 10–110 mV s^−1^. The same linear range can be observed for the reduction peak at 0.22 V, which slightly shifts to the right. The linear relationship of the square root of the scan rate with the values of the anodic peak current (I_pa_) and cathodic peak current (I_pc_) can be seen in Figure 3b. 

Taking into account the linear regression Equations (4) and (5), for seven data points, below, the influence of scan rate explains the electrode process in terms of a diffusion controlled reaction (mass transport):

I_pa_ (µA) = 0.48 v^1/2^ + 1.21 (mV s^−1^)^1/2^, R^2^ = 0.969 (4)

I_pc_ (µA) − =1.15 v^1/2^ − 2.59 (mV s^−1^)^1/2^, R^2^ = 0.987 (5)

### 3.2. Antioxidant Properties of the Plant Extracts

#### 3.2.1. Scanning Electron Microscopy

**Figure 4:**
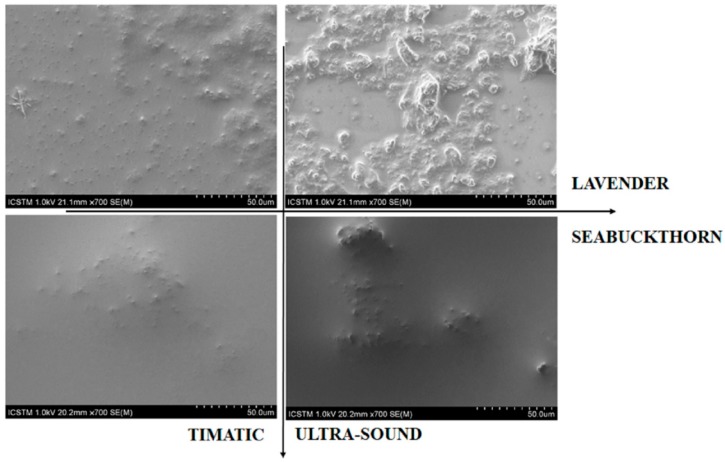
Scanning electron micrographs of 1.Lf Aa T and 1.Hf Aa T on the left and 1.Lf Aa US and 1.Hf Aa US on the right.

Scanning electron micrographs of four types of extracts are shown in Figure 4 and provide an overview of their morphological structure. In a previous work [30], it has been shown that all extracts obtained through pressure enhanced solvent extraction (Timatic) have a higher content of phenolics (for example 2.Lf Ab T has a total of phenolics of 31.97 µg /mg total extract in comparison to 2.Lf Ab US, which has 11.43 µg /mg total extract). A smoother morphology is shown here and therefore a better dispersion of the material. For lavender extracts, ultra-sound assisted extraction was shown to isolate less phenolic compounds in comparison to pressure assisted extraction [30], scanning electron micrographs revealing an agglomeration of insolubilized compounds. Both lavender extracts show a higher agglomeration of compounds in comparison to sea buckthorn extracts. There could be a correlation between the morphology of the extract and their AC, depending on the extraction method.

#### 3.2.2. Electrochemical Evaluation of Relative Antioxidant Capacity

Electrochemical methods for AC detection are a more rapid and simple alternative towards classical methods. There are several parameters which can be determined using cyclic voltammetry (anodic current peaks, peak potentials and the area under the anodic wave-Q) which help characterize the AOx compounds present in different samples [31]. This is possible due to the reducing power of AOx present in our hydrosoluble plant extracts. Certain molecules act as electron or proton donors in redox reactions, having the ability to reduce (quench) reactive species such as H_2_O_2_.

For this purpose we optimized a simple screening method for rapid plant extract characterization through relative antioxidant capacity determination using H_2_O_2_ label-free electrochemical sensors. The sensor material has an important role upon the sensor response. Based on the results obtained during electrochemical sensor characterization, AuNP-based electrodes were used, further demonstrating the synergy between nanotechnology and electrochemical techniques.

##### Cyclic Voltammetry

**Figure 5:**
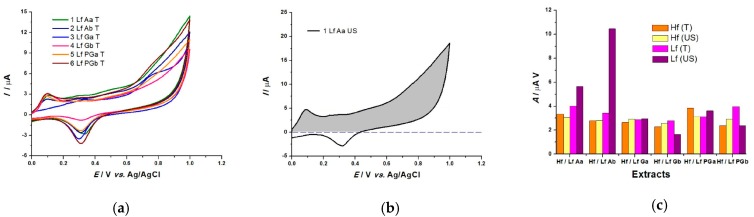
(**a**) Cyclic voltammograms, sweeping potential 0-1V, in 0.1 M NaPB, pH = 7.0, ν = 50 mV s^−1^ for all pressure extracted lavender extracts; (**b**) Calculation of the anodic area and (**c**) Histogram showing all areas.

As mentioned before, CV measures a variety of parameters to analyze the AC of our extracts. It was shown that peak potentials reflect the redox properties, while current intensity and anodic area values describe the charge transferred during the scavenging process. Figure 5a illustrates the CV profile between 0–1 V, in 0.1 M NaPB, pH = 7.0, ν = 50 mV s^−1^ of the lavender extracts obtained through pressure extraction, revealing a pronounced anodic peak at 0.09 V, and a small peak at 0.35 V present for only some of the extracts. It was shown [28] that anodic peaks at low oxidation potentials (below 0.45 V) occur for compounds with significant antioxidant capacity.

Further, the anodic area of a CV can be correlated with the reducing power of the extracts, and herewith, with their AC [32]. The previously recorded voltammograms were used to calculate the anodic area (greyed out) as shown in Figure 5b. Figure 5c represents the AC calculated from the anodic area of the voltammograms for all lavender and sea buckthorn extracts after subtracting the anodic area of the electrode in the absence of the extract.

##### Amperometry-H_2_O_2_ Sensor for Relative Antioxidant Capacity

**Figure 6:**
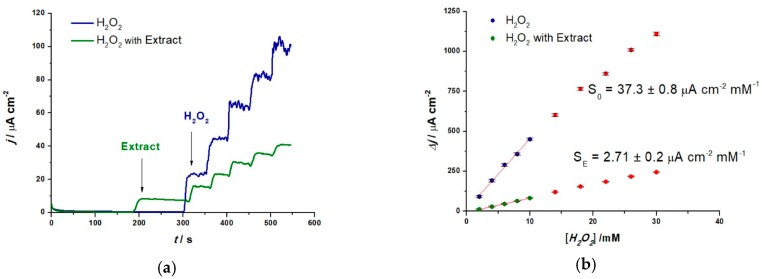
(**a**) Fixed potential amperometry at 0.55 V vs. Ag/AgCl for H_2_O_2_ sensor in the presence and absence of 2.Hf Ab US extract in 0.1 M NaPB, pH = 7.0; (**b**) Corresponding calibration plots.

Amperometry is often used in AC detection since it allows the evaluation of current obtained for a fixed working potential. The current intensity varies for different products and can be easily correlated to results obtained with spectrophotometry [33]. Since different methods make use of different chemicals, RAC is often expressed as an equivalent towards a standard, such as ascorbic acid [23] or Trolox [34]. Fixed potential amperometry was used in order to determine the RAC of the extracts. This was done using AuNP based label-free electrochemical sensors for H_2_O_2_ by monitoring their scavenging in the presence of extracts. This way the RAC can be determined using the equation below:

RAC (%) = [(S_0_ – S_E_)/S_0_] × 100 (6)

where S_0_ represents the sensor sensitivity for H_2_O_2_ in the absence of the extract, and S_E_ the sensitivity in the presence of the extract [35]. To be able to calculate the relative antioxidant capacity of each extract, first, the H_2_O_2_ AuNP sensor was evaluated in the absence of the extract. The calibration plots were constructed from amperometric measurements (n = 3) performed in 0.1 M NaPB (pH 7.0), at 0.55 V vs. Ag/AgCl applied potential, with increasing H_2_O_2_ concentrations from 2 to 30 mM, as shown in Figure 6a. The value of the fixed potential represents the oxidation potential of H_2_O_2_ and was chosen from the data obtained during CV. In the absence of an extract, a sensitivity of S_0_ = 37.29 ± 0.87 µA cm^−2^ mM^−1^ (n = 3) was obtained. After adding an extract (1 : 40 extract:buffer dilution), for example 2. Hf Ab US, a much lower sensitivity was obtained, illustrating the scavenging effect of the AOx present in the extract towards H_2_O_2_, as shown in Figure 6b.

**Figure 7:**
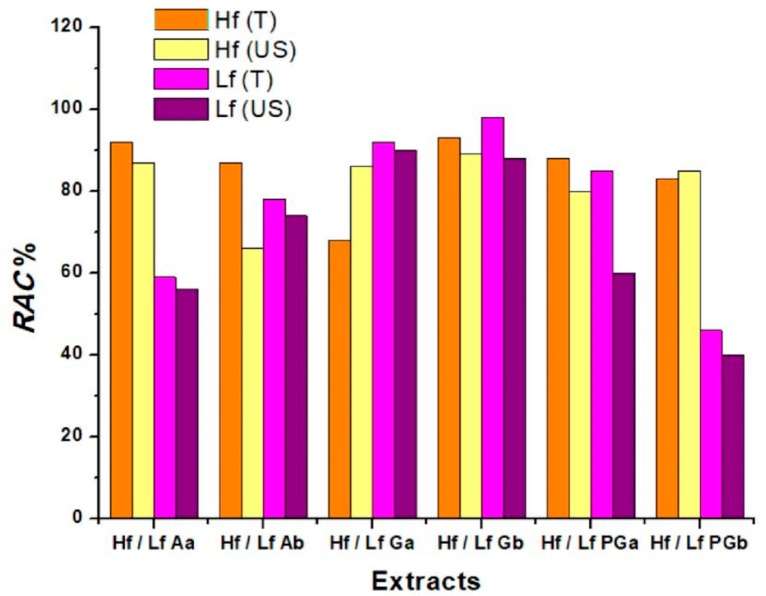
Histogram for the relative antioxidant capacity of each extract calculated using Equation (6).

Figure 7 shows the RAC values for lavender and sea buckthorn extracts, obtained using the amperometric H_2_O_2_ sensor and calculated with Equation (6). The lowest sensitivities, highest RAC, suggest the highest scavenging property of the extract. We can also see that the influence of solvents as well as the influence of the extraction method brings changes towards the antioxidant capacity of each plant extract.

**Figure 8:**
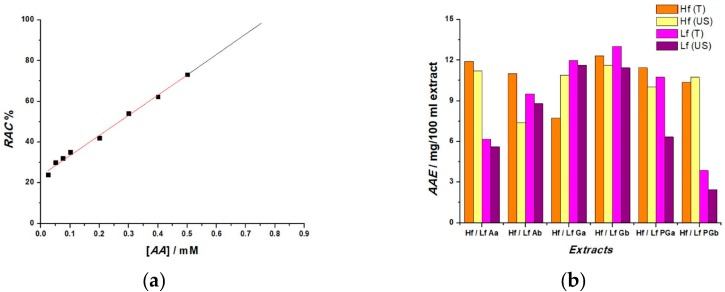
(**a**) Calibration plot for increasing AA concentrations and 0.5 mM H_2_O_2_ obtained from fixed potential amperometry at 0.55 V vs. Ag/AgCl; (**b**) Histogram RAC expressed in terms of AAE.

For comparison reasons and a more accurate expression of RAC, its values can be expressed in terms of ascorbic acid equivalent (AAE). Therefore, for each increasing, fixed concentration of AA (0.025 to 0.5 mM) a calibration plot for the H_2_O_2_ sensor was constructed. Using Equation (6), the RAC value for each AA concentration was calculated and the results were plotted as a function of AA concentrations, as shown in Figure 8a. The linear region was extrapolated, and the RAC values obtained for the extracts were identified and associated with the corresponding AA concentrations expressed in mM and mg/100 mL solvent allowing the calculation of AAE for the extracts as shown in Figure 8b.

**Figure 9:**
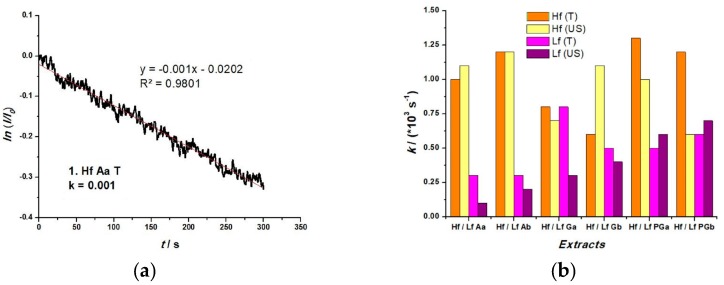
(**a**) Relative current decay in semi-logarithmic plot; (**b**) Histogram of the kinetic constant of H_2_O_2_ inhibition for all extracts.

The antioxidant activity of the extracts can be estimated based on the kinetics of H_2_O_2_ scavenging in the presence of an antioxidant compound from the extract (competitive inhibition). This method was proposed by Karyakina et al. [36] and is also based on an amperometric H_2_O_2_ sensor. For this purpose, amperometric measurements were performed for each extract:buffer solution at 0.55 V vs. Ag/AgCl. Once a stable baseline was acheived, a 0.5 mM H_2_O_2_ sample solution was added to the electrolyte. After achieving a maximum response, the kinetics of the current decay was investigated. AuNPs easily catalyse H_2_O_2_ oxidation, generating an anodic response. Thus, we can be sure that the decay of anodic current is due to H_2_O_2_ scavenging. The current decay is assumed to obey the pseudo-first order kinetics [35]. Since the semi-logarithmic plots do not display a perfect linearization over the whole time interval, an initial slope over 300 seconds was taken into consideration. The kinetic constant (k) of H_2_O_2_ inhibition, can be determined from the slope in the semi-logarithmic plots of the ratio between the current intensity of H_2_O_2_ in the absence (I_0_) and presence (I) of the extract, as shown in Figure 9a. Figure 9b presents the values of the kinetic constant for the extracts, being a relative indicator of the antioxidant activity of the extracts. The higher the value of k, more powerful antioxidant compounds are present in the extract.

There is a difference between antioxidant activity and antioxidant capacity. While the antioxidant activity is related to the kinetics of the reaction between an oxidant and an antioxidant, the antioxidant capacity refers to the conversion efficiency of the ROS being scavenged. It yields the amount, in moles, of the scavenged free radical by antioxidants [37,38], in our case represented by the analyzed plant extracts.

#### 3.2.3. Statistical Analysis

In order to validate the results obtained through electrochemical methods, correlations to classical methods are necessary. However, it must be taken into account that different techniques use a variety of solutions and chemicals, giving herewith different results. UV-Vis spectrophotometry and chemiluminescence were correlated with the electrochemical amperometric measurements. Measurements were done in triplicate and expressed as means ± their standard deviation (SD). Statistical analysis was performed using paired *t*-test, while correlations among data were calculated and the correlation coefficient was calculated using Pearson’s correlation:

##### Spectrophotometry

**Figure 10:**
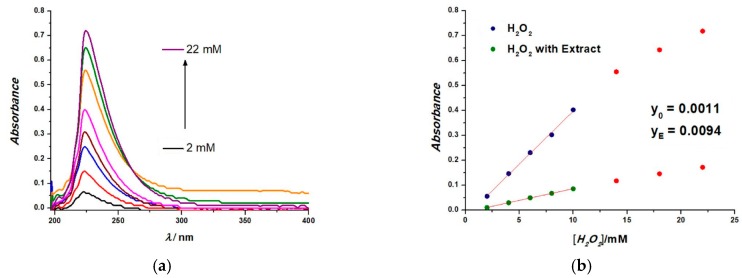
(**a**) Absorbance spectra at λ = 225 nm for successive H_2_O_2_ addition; (**b**) Calibration plots obtained for increasing H_2_O_2_ addition in the presence and absence of 1.Hf Aa US in 0.1 M NaPB, pH = 7.0, from the absorbance spectra.

Similar to electrochemical RAC detection, UV-Vis absorbance spectra can be used to characterize the oxidation process of H_2_O_2_. Classical UV-Vis spectrophotometry allows direct measurement of different H_2_O_2_ concentrations through absorbance at 225 nm, or through the reaction of H_2_O_2_ molecules with phenolic compounds [39]. Here, next to the lavender and sea buckthorn extracts, we also analyzed the hydroalcoholic extracts with water “a” for walnut (Js) and grape (Vp) extracts, as shown in Table 2. The characterization of H_2_O_2_ oxidation in the absence and presence of an extract were monitored in a 2-mL cuvette containing a 1:40 extract:buffer solution, with increasing H_2_O_2_ addition. The absorption spectra were recorded for 200 < λ < 400 nm. We observed a slight shift towards right of the absorbance peaks for H_2_O_2_ (from 240 nm to 250 nm) depending on each extract. Figure 10a shows the absorbance spectra for H_2_O_2_, while Figure 10b shows the calibration plot for H_2_O_2_ in the absence and presence of 1.Hf Aa US calculated from the absorption spectra at λ = 225 nm.

RAC can be calculated in the same manner as for electrochemical methods, based on H_2_O_2_ inhibition in the presence of an extract, using the equation below:

RAC (%) = 1 − (y_E_/y_0_) × 100 (7)

where y_0_ is the slope of the calibration plot for H_2_O_2_ (blank) and y_E_ is the slope of the calibration plot in the presence of the extract [32].

Table 3 presents the calculated RAC (%) values of lavender and sea buckthorn extracts, in comparison with the other two plant extracts obtained using the same extraction methods, for both electrochemical and spectrophotometric methods. Data are expressed as the mean of triplicate measurements ± SD. The antioxidant capacity of the extracts varied between 20% and 84.5%, depending on solvent and extraction method. Applying the paired *t*-test in order to prove that our two data sets are not significantly different, we obtained the calculated value t = 2.066, for seven degrees of freedom. For P = 0.05, the critical value t_7_ = 2.36 (reading from the tables of the t-distribution [40]). Since the calculated value of |t| is less than the critical one, the null hypothesis is not rejected. Using Pearson’s correlation on the same data set, a correlation coefficient of r = 0.907 was obtained, which indicates a high positive correlations among the two methods.

##### Chemiluminescence

The chemistry of chemiluminescent determination of antioxidants is based on the reaction of ROS or RNS with chemiluminescent reagents to produce species in an excited state that lights up. The antioxidants react with the initiating reactive species and inhibit light production. Hence, generally, chemiluminescence methods for antioxidant capacity determination are based on competitive reactions [37].

For chemiluminescence measurements, the following assay was used: in an Eppendorf tube of 2 mL, 350 µL of working solution for CL determinations (10^−3^ M Na_2_EDTA, 0.8 × 10^−3^ M CoCl_2_, 1.1 × 10^−4^ M luminol), 350 µL of 0.1 M, pH = 9.0 borate buffer solution and 350 µL of 3 × 10^−4^ M H_2_O_2_ solution were introduced. The mixture was homogenized. The chemiluminescence signal reached a plateau approximately 600 seconds after the start of measurements, then the intensity of chemiluminescent radiation was measured and denoted as I_0_. Afterwards 25 µL of the analyzed sample (antioxidant standard or plant extract solutions) were added to the reaction mixture which was homogenized. A sudden decrease of the CL signal is registered. This value is denoted as I. 

**Figure 11:**
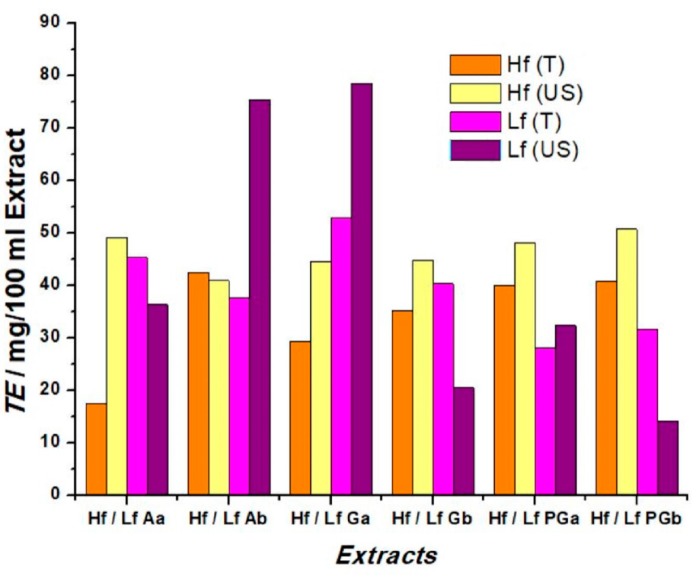
Histogram of CL determinations in terms of Trolox equivalent, for lavender and sea buckthorn extracts.

The computed I_0_/I ratio was then plotted as a function of Trolox, following the procedure described in [41] (Supplementary Materials). The same procedure was used for plant extract analysis and the results are shown in Figure 11; where the Trolox equivalent (TE) was calculated for all analyzed extracts. The intensity ratio for each extract was associated to the mass of dry plant/100 mL solvent allowing the calculation of TE. In order to be able to correlate the data obtained using chemiluminescence and electrochemistry, the RAC values were expressed in terms of Trolox equivalents, TE, in the same manner as it was done for AAE. The obtained values, shown in Table 4, represent the RAC values expressed in TE (mg/100 mL extract) for some of the extracts for both electrochemical and classical chemiluminescence methods.

**Figure 12:**
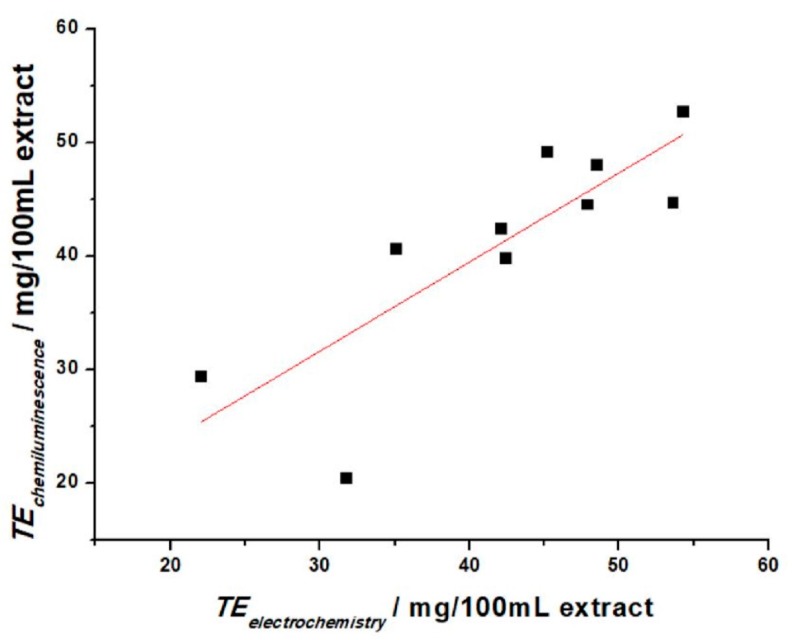
Correlation between electrochemical and chemiluminescence TE values. Correlation coefficient: r = 0.822.

Depending on the type and activity of antioxidant components in the extracts, TE values vary from 20.5 to 54.2 mg/100 mL extract, and are comparable between the two methods. Applying the paired *t*-test, we obtained the calculated value t = 0.521, for nine degrees of freedom. For P = 0.05, the critical value t_9_ = 2.26 (reading from the t-distribution tables [41]). Since the calculated value of |t| is significantly less than the critical one, we can say that the two methods are not significantly different. Using Pearson’s correlation on the same data set, a correlation coefficient of r = 0.822 was obtained, which indicates a positive correlation among the two methods. This lower value can be explained due to the significant differences in the two methods, where chemiluminescence makes use of several chemicals and markers. The scatter plot in Figure 12 shows how the data for the two methods follow a linear tendency, in accordance with the value of the correlation coefficient.

## 4. Conclusions

This article focuses on the development and optimization of nanoparticle-based H_2_O_2_ label-free electrochemical sensors for antioxidant capacity determination of hydrosoluble plant extracts using their H_2_O_2_ scavenging properties due to the presence of antioxidant compounds. Several antioxidant compounds were found in hydrosoluble extracts of lavender, sea buckthorn, grapes and walnuts. Ultrasound-assisted and pressure enhanced solvent extraction at 6.7 bar, as well as different solvents, were used to isolate the active compounds, whose relative antioxidant capacity was analyzed electrochemically, and correlated to classical methods. AuNP modified sensors showed the best catalytic activity towards H_2_O_2_ scavenging compared with graphene and carbon nanotube-based ones, being used throughout all measurements.

Using CV, several parameters were investigated in the analysis of AC in the extracts. The peak potentials reflect the redox properties, where the peaks for some of the lavender and sea buckthorn extracts are more pronounced. The values obtained from the anodic area describe the charge transferred during the scavenging process, where a high AC was registered for 1.Lf Aa US (5.65 µAV) and 2.Lf Ab US (10.45 µAV). The antioxidant activity of the extracts can be estimated based on the kinetics of H_2_O_2_ scavenging in the presence AOx. In this case, sea buckthorn extracts had a higher scavenging activity (most of the k values being above 1 × 10^3^ s^−1^) in comparison to lavender extracts (the highest value of k = 0.8 × 10^3^ s^−1^).

The optimized AuNP-based sensor for H_2_O_2_ was used to highlight the relative antioxidant capacity of the extracts. The most powerful antioxidants were found in sea buckthorn and lavender extracts with the highest RAC of 98% for 4.Lf Gb T. For comparison reasons RAC values were converted and expressed in terms of AAE and TE (mg/100 mL extract). We found a good correlation of electrochemical and spectrophotometric RACs, while the correlation coefficient between electrochemical and chemiluminescence TE was lower (0.822). This was attributed to the differences between methods. Considering these results, the optimized AuNP-based label-free electrochemical sensor for H_2_O_2_ can be used as a simple, rapid alternative towards classical methods for RAC detection of hydrosoluble plant extracts.

## Figures and Tables

**Table 1:** List of used plant extracts with corresponding extraction solvent and extraction method.

Solvent Type	*Hippophaefructus* Extracts	*Lavandula* Flowers Extracts
Hydroalcoholic solution (1:1) water “a”: ethanol	1.Hf Aa T / 1.Hf Aa US	1.Lf Aa T / 1.Lf Aa US
Hydroalcoholic solution (1:1) water “b”: ethanol	2.Hf Ab T / 2.Hf AbUS	2.Lf Ab T / 2.Lf Ab US
Hydroglycerine solution (1:1) water “a”: glycerin	3.Hf Ga T / 3.Hf Ga US	3.Lf Ga T / 3.Lf Ga US
Hydroglycerine solution (1:1) water “b”: glycerin	4.Hf Gb T / 4.Hf Gb US	4.Lf Gb T / 4.Lf Gb US
Hydropropyleneglycol solution (1:1) water “a”: propylene glycol	5.Hf Pga T / 5.Hf Pga US	5.Lf Pga T / 5.Lf Pga US
Hydropropyleneglycol solution (1:1) water “b”: propylene glycol	6.Hf Pgb T / 6.Hf Pgb US	6.Lf Pgb T / 6.Lf Pgb US

**Table 2:** List of used hydroalcoholic plant extracts with corresponding extraction method used in spectrophotometry in addition to the extracts in Table 1.

Solvent Type	Juglans Regia Extracts	Vitis ViniferaExtracts
Hydroalcoholic solution (1:1) water “a”: ethanol	1.Js Aa T / 1.Js Aa US	1.Vp Aa T / 1.Vp Aa US

**Table 3:** RAC (%) for alcoholic plant extracts.

Extracts	Electrochemical RAC (%)	Spectrophotometry RAC (%)
1.Lf Aa US	83.8 ± 0.04	77.6 ± 0.11
1.Hf Aa US	72.8 ± 0.22	79.0 ± 0.09
1.Js Aa US	30.9 ± 0.32	56.7 ± 0.92
1.Vp Aa US	47.6 ± 0.83	73.4 ± 1.08
1.Lf Aa T	78.5 ± 0.03	84.5 ± 0.07
1.Hf Aa T	69.6 ± 0.20	81.9 ± 0.24
1.Js Aa T	20.0 ± 0.44	33.3 ± 1.05
1.Vp Aa T	36.7 ± 0.23	58.2 ± 2.11

**Table 4:** TE equivalent for some of the lavender and sea buckthorn extracts.

Extracts	Electrochemistry TE (mg/100 mL extract)	Chemiluminescence TE (mg/100 mL extract)
1.Hf Aa US	45.1 ± 0.22	49.2 ± 0.90
2.Hf Ab T	42.0 ± 0.85	42.5 ± 1.40
3.Hf Ga T	22.0 ± 0.90	29.4 ± 1.00
3.Hf Ga US	47.8 ± 0.36	44.6 ± 0.82
4.Hf Gb US	53.5 ± 1.28	44.8 ± 3.90
5.Hf PGa T	42.3 ± 0.78	39.9 ± 1.50
5.Hf PGa US	48.4 ± 0.36	48.1 ± 0.80
6.Hf PGb T	35.0 ± 0.60	40.7 ± 1.50
3.Lf Ga T	54.2 ± 1.05	52.8 ± 4.60
4.Lf Gb US	31.7 ± 0.50	20.5 ± 0.84

## Supplementary Materials

The following are available online at http://www.mdpi.com/1424-8220/19/3/590/s1, Figure S1: CV in 0.1 M NaPB, pH 7.0, in the presence of 6 mM H_2_O_2_ addition, v = 50 mV s^−1^ for bare SPE (C) and nanoparticle modified SPE with AuNP, CNT and G.; Table S1: Capacitance values calculated for all sensor types from the CVs in Figure S1; Figure S2: Calibration plots recorded at 0.55 V vs. Ag/AgCl in 0.1 M NaPB with an E for different dilutions, pH = 7.0., Table S2: Performance parameters for the three different E:buffer dilutions in Figure S2, Figure S3: Calibration plot of CL signal (I0/I) as a function of Trolox concentrations.
