# Supplementary material for: A Nanoparticle-Based Label-Free Sensor for Screening the Relative Antioxidant Capacity of Hydrosoluble Plant Extracts
Source: Sensors (Basel). 2019 Jan 30;19(3):590. doi: 10.3390/s19030590 (PMC6387368; doi:10.3390/s19030590)
Supplement: Supplementary file 1 [file sensors-19-00590-s001.pdf]

sensors

MDPI

*Supplementary Materials*

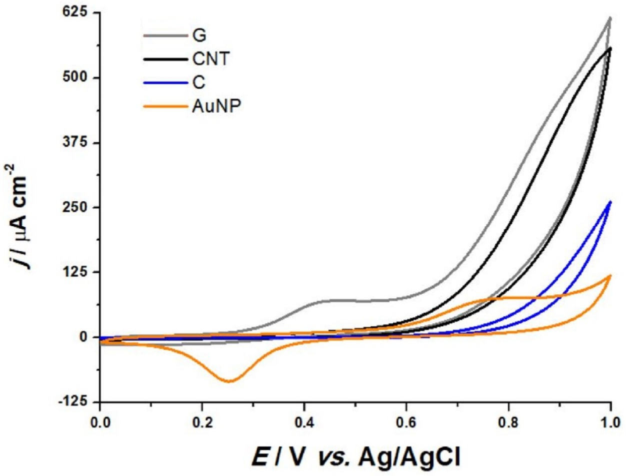


**Figure S1.** CV in 0.1 M NaPB, pH 7.0, in the presence of 6 mM $H_2O_2$ addition, v = 50 mV $s^{-1}$ for bare SPE (C) and nanoparticle modified SPE with AuNP, CNT and G.

**Table S1.** Capacitance values calculated for all sensor types from the CVs in figure S1.

| Sensor (SPE) | C/mF cm$^{-2}$ |
|---|---|
| bare (C) | 0.03 |
| G | 1.23 |
| CNT | 0.30 |
| AuNP | 0.34 |

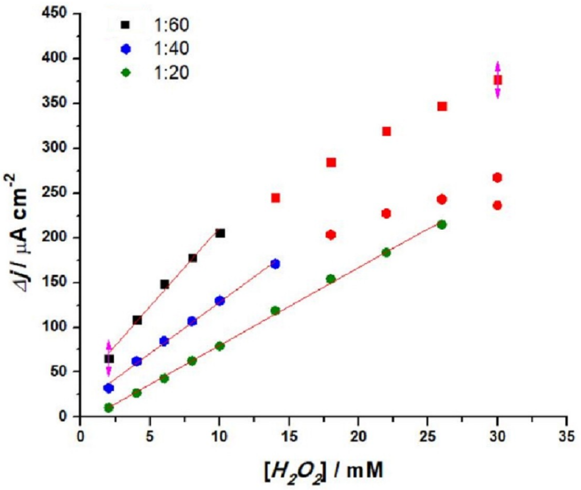


**Figure S2.** Calibration plots recorded at 0.55 V vs. Ag/AgCl in 0.1 M NaPB with an extract for different dilutions, pH = 7.0.

**Table S2.** Performance parameters for the three different E: buffer dilutions in figure S2.

| Extract | S/mA mM$^{-1}$ cm$^{-2}$ | $R^2$ | LoD/mM |
|---|---|---|---|
| 1:20 | 8.66 | 0.998 | 0.60 |
| 1:40 | 11.37 | 0.995 | 0.75 |
| 1:60 | 17.46 | 0.991 | 1.76 |

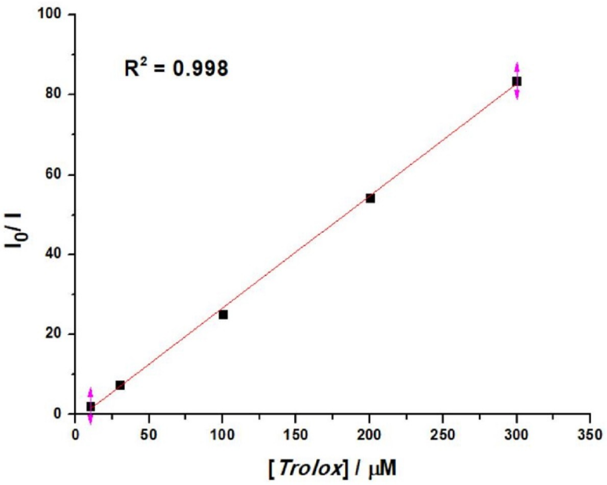


**Figure S3.** Calibration plot of CL signal ($I_0$/I) as a function of trolox concentrations.

© 2018 by the authors. Submitted for possible open access publication under the terms and conditions of the Creative Commons Attribution (CC BY) license (http://creativecommons.org/licenses/by/4.0/).
